# Safety of Laparoscopic Hernia Surgery in Patients With Preoperative Antiplatelet Continuation Therapy

**DOI:** 10.7759/cureus.82287

**Published:** 2025-04-15

**Authors:** Keiji Nagata, Takahisa Fujikawa

**Affiliations:** 1 Surgery, Kokura Memorial Hospital, Kitakyushu, JPN

**Keywords:** antiplatelet therapy, antithrombotic therapy, aspirin monotherapy, bleeding complications, perioperative management

## Abstract

Introduction

The optimal perioperative antithrombotic management of patients receiving antithrombotic therapy (ATT) remains controversial. In this study, we investigated the safety and feasibility of laparoscopic hernia surgery in patients taking ATT, especially those with a preoperative continuation of single antiplatelet therapy (APT).

Methods

Three hundred ninety-six (396) patients who underwent laparoscopic hernia surgery between April 2014 and March 2023 in our institution were retrospectively reviewed. The patients were divided into two groups: patients who continued single aspirin monotherapy preoperatively (continued single aspirin therapy (cAPT) group; n = 118) and patients who did not receive APT preoperatively (non-APT group; n = 278). Our perioperative antithrombotic management included preoperative continuation of single aspirin therapy for patients with APT or interruption of oral anticoagulation therapy (ACT), bridging anticoagulation with unfractionated heparin or direct-acting oral anticoagulants (DOAC) replacement for patients with ACT. The primary outcome was postoperative bleeding complications (BC).

Results

There were four postoperative BCs (Clavien-Dindo classification ≧ Ⅱ) (1.0%) in the whole cohort, one (0.9%) in the cAPT group, and three (1.1%) in the non-APT group, which were not significantly differentiated (p = 0.8330). Multivariable analysis showed heparin or DOAC replacement was an independently and significantly risk factor for postoperative bleeding (p = 0.0029, odds ratio (OR) = 32.6). Continuation of preoperative aspirin was not a risk factor for postoperative BCs. No thromboembolic complications occurred in the whole cohort.

Conclusion

We can safely and feasibly perform laparoscopic hernia surgery under preoperative antithrombotic management, including the preoperative continuation of single aspirin therapy, without any increase in bleeding events. However, careful consideration is required for the patient who received heparin bridging or DOAC replacement.

## Introduction

As the number of patients with cardiovascular or cerebrovascular diseases rises in an aging society, more patients undergo antithrombotic therapy (ATT), which includes antiplatelet therapy (APT) and/or anticoagulation therapy (ACT), for secondary prevention of these conditions [1,2]. Consequently, the number of patients referred for surgical treatment and receiving ATT is also growing.

Perioperative management of these patients is contentious among surgeons since they are frequently at high risk of bleeding or thromboembolic consequences. The risk of bleeding complications (BC) may increase if ATT is sustained; however, the risk of thromboembolic complications (TC), such as acute myocardial infarction, coronary stent thrombosis, and cerebral infarction, may increase if ATT is discontinued during the perioperative period. Since TC can have fatal or serious sequelae once it has happened [3,4], it is more crucial to prevent TC than to prevent BC. For these reasons, recent guidelines concerning antithrombotic management recommend that for patients receiving APT who are at high risk of thromboembolism, we should continue APT, or at least maintain a single aspirin therapy, throughout the perioperative period [2,5]. However, most institutions prefer to discontinue APT during the perioperative period for digestive surgeries that carry bleeding risks. Moreover, a large number of laparoscopic hernia surgeries have been performed, and the number is expected to increase further.

This study aims to assess the safety and feasibility of laparoscopic hernia surgery in patients undergoing APT.

## Materials and methods

We retrospectively reviewed a total of 396 patients who underwent laparoscopic hernia surgery for inguinal hernia or ventral hernia in our institution between April 2014 and March 2023. The treatment strategy for inguinal hernia and ventral hernia surgery in our hospital is laparoscopic surgery as the first choice, except for the following conditions: a history of radical prostatectomy, patients who have multiple previous lower abdominal open surgeries and are expected to have severe adhesions in the abdominal cavity, and patients whose physical status (PS) is too inadequate to tolerate general anesthesia. We excluded cases of emergency surgery and those with insufficient information in the medical records.

**Figure 1:**
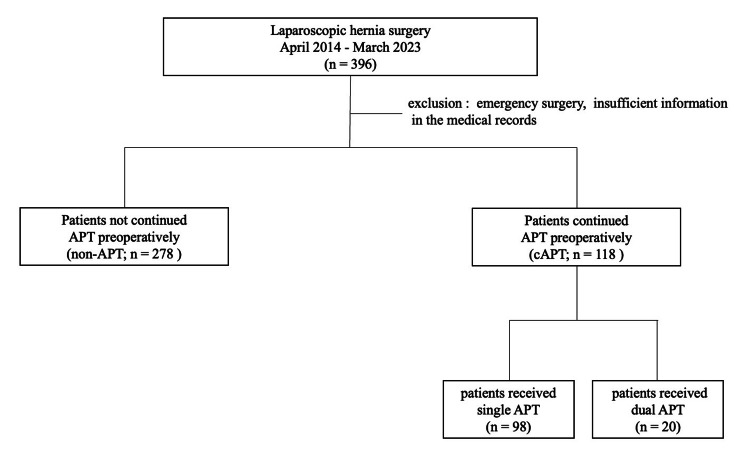
Flowchart of this study A total of 396 patients who underwent laparoscopic hernia surgery were divided into two groups based on the status of preoperative APT. APT: antiplatelet therapy, cAPT: continued single aspirin therapy

Patients were divided into two groups based on their preoperative APT status: those who received continued single aspirin therapy (cAPT) (cAPT group; n = 118) and those who did not receive continued APT (non-APT group; n = 278) (Figure 1). There were 21 cases in low-thromboembolic-risk patients in which APT was discontinued five to seven days before surgery. These cases were assigned to the non-APT group. There were 20 cases of high-thromboembolic-risk patients who received dual antiplatelet therapy (DAPT); only aspirin was continued, while the other drug was discontinued seven days before surgery. These cases were included in the cAPT group.

A standardized evaluation of the electronic surgical database, along with hospital and clinical records, was used to collect patient characteristics, perioperative variables, and postoperative outcomes of the included patients. The condition of patients’ functions and symptoms concerning the requirement for care and their ambulatory status were described using the American Society of Anesthesiologists Physical Status Classification (ASA-PS). Postoperative complications were evaluated and classified using the Clavien-Dindo classification (CDC) [6], with CDC class Ⅱ or higher indicating significance. Postoperative BC was diagnosed as bleeding or hematoma, including intraperitoneal bleeding, subcutaneous hemorrhage, and abdominal wall hematoma formation, as confirmed by computed tomography (CT) or ultrasonography. In addition, postoperative bleeding with CDC class Ⅱ or higher was defined as the primary outcome. We compared background characteristics, perioperative conditions, and outcome variables between these groups. All procedures were performed by or under the guidance of one of the board-certified attending surgeons in our institution.

Perioperative antithrombotic management 

**Figure 2:**
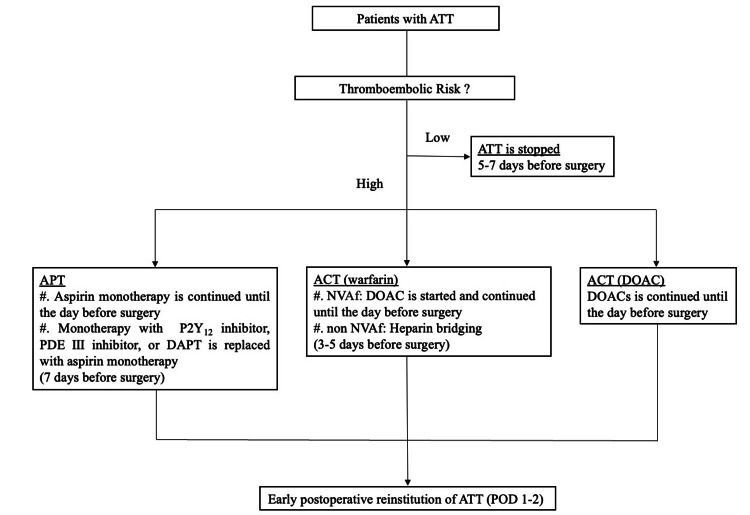
Perioperative antithrombotic management protocol (“Kokura Protocol”) for patients with ATT in the case of elective surgery ACT: anticoagulation therapy, APT: antiplatelet therapy, ATT: antithrombotic therapy, DAPT: dual antiplatelet therapy, DOAC: direct-acting oral anticoagulant, NVAF: nonvalvular atrial fibrillation, PDE: phosphodiesterase, POD: postoperative day

We have established our own perioperative antithrombotic management protocol, known as the “Kokura Protocol,” that comprises thromboembolic risk assessment. We have also shown that patients with ATT can safely perform both open and laparoscopic abdominal surgeries under the Kokura Protocol [7,8]. The perioperative flowchart for patients with ATT under the Kokura Protocol is shown in Figure 2.

Management generally discontinues ATT five to seven days before surgery and postoperative early reinstitution in low-thromboembolic-risk patients. We continue preoperative aspirin monotherapy at a dose of 100 mg/day for high-thromboembolic-risk patients who receive APT until the day before surgery. Monotherapy with a thienopyridine P2Y12 inhibitor or phosphodiesterase (PDE) Ⅲ inhibitor, or dual APT (DAPT) with aspirin plus a P2Y12 inhibitor or a PDE Ⅲ inhibitor, is replaced with aspirin monotherapy (100 mg/day) seven days before surgery. For patients who receive warfarin and have a high risk of thromboembolic events, the management included interruption of warfarin, bridging anticoagulation with unfractionated heparin, or direct-acting oral anticoagulants (DOAC) three to five days before surgery and continued until the day before surgery. In patients with DOAC, DOAC is continued until the day before surgery, modifying it if there is a decrease in renal function. Every antithrombotic medication is resumed as soon as feasible postoperatively (POD1-2) when hemostasis is confirmed.

Patients with the following characteristics were defined as having high-thromboembolic risks: (1) patients undergoing coronary stenting; (2) patients undergoing cerebrovascular reconstruction within three months; (3) patients having a recent-onset cerebral stroke or transient ischemic attack; (4) patients with regular oral anticoagulation for chronic atrial fibrillation or venous thrombosis, including pulmonary embolism or deep venous thrombosis; and (5) patients having cardiovascular or cerebrovascular diseases who were recognized as having high risk for thromboembolism by cardiac/cerebral specialists. 

Statistical analysis 

The categorized data were compared by χ^2^ (chi-square) or Fisher’s exact probability test. Continuous variables in the patient characteristics were expressed as a median with an interquartile range and compared by the Wilcoxon signed-rank test. Nonparametric variables were also compared by using the Wilcoxon signed-rank test or the Student’s t-test. Univariate and multivariate logistic regression analyses were used to determine risk factors influencing postoperative BCs. Only variables with p < 0.05 in the univariate analysis were considered significant and were included in the subsequent multivariate analysis. Statistical significance was set at p < 0.05. The data were statistically analyzed using the JMP software version 10.0.2 (SAS Institute Inc., Cary, NC).

## Results

Patient characteristics of this study

A total of 396 patients underwent laparoscopic hernia surgery between April 2014 and March 2023. One hundred eighteen patients (29.8%) had received cAPT preoperatively (cAPT group), whereas 278 patients (70.2%) had not received APT preoperatively or discontinued APT five to seven days before surgery (non-APT group). Table 1 shows the patient characteristics of each group in this study.

**Table 1:** Patient characteristics of this study ASA-PS: American Society of Anesthesiologists-Physical Status, BMI: body mass index, CABG: coronary artery bypass graft, cAPT: continued single aspirin therapy, PCI: percutaneous coronary intervention, TIA: transient ischemic attack

Factors	Total (n = 396)	cAPT (n = 118)	non-APT (n = 278)	p-value
Age, years, median (range)	74 (67-79)	76 (69-81)	73 (66-78)	0.0014
Male gender, n (%)	347 (88)	104 (88)	243 (87)	0.8411
BMI, kg/m^2^, median (range)	23.1 (21.1-25.2)	22.5 (21.0-24.9)	23.3 (21.2-25.3)	0.4115
ASA-PS ≧ 3, n (%)	94 (24)	45 (38)	49 (18)	<0.0001
Concurrent diseases: Hypertension, n (%)	265 (67)	99 (84)	166 (60)	<0.0001
Concurrent diseases: Diabetes mellitus, n (%)	56 (14)	21 (18)	35 (13)	0.1738
Concurrent diseases: Hx of cerebral infarction/TIA, n (%)	59 (15)	34 (29)	25 (9)	<0.0001
Coronary artery disease: Hx of PCI, n (%)	92 (23)	76 (64)	16 (6)	<0.0001
Coronary artery disease: Hx of CABG, n (%)	11 (3)	8 (7)	3 (1)	0.0016
Regular hemo/peritoneal dialysis, n (%)	20 (5)	4 (3)	16 (6)	0.3255
CHA_2_DS_2_-VASc score ≧ 2, n (%)	322 (81.3)	117 (99.2)	205 (73.7)	<0.0001
CHADS_2_ score ≧ 2, n (%)	221 (56)	94 (80)	127 (46)	<0.0001

In the cAPT group, poor ASA-PS scores (class 3; a patient with severe systemic disease) or (4; a patient with severe systemic disease that is a constant threat to life) were observed, and the occurrences of underlying diseases, including the history of hypertension, cerebral infarction/transient ischemic attack (TIA), percutaneous coronary intervention (PCI), and coronary artery bypass graft (CABG), were higher. The high-risk category, according to the CHADS2 scoring system [9] and the CHA_2_DS_2_-VASc scoring system [10], was observed in the cAPT group. The CHADS_2_ (congestive heart failure (CHF), hypertension, age ≥75 years, diabetes, stroke/TIA) score and the CHA_2_DS_2_-VASc (CHF, hypertension, age ≥75 years, diabetes, stroke/TIA, vascular disease, age 65-75 years, and sex category) score are the widely used scoring systems to assess the risk of stroke in patients suffering from atrial fibrillation. A high score corresponds to a greater risk of stroke, while a low score corresponds to a lower risk of stroke. Such a score is used to determine whether or not treatment is required with ACT or APT.

Factors concerning operative procedures and postoperative morbidity

Table 2 displays the operative procedures, perioperative characteristics, and postoperative morbidity in each group. The cohort study performed a transabdominal preperitoneal approach (TAPP) on 311 patients, totally extraperitoneal approach (TEP) on 45 patients, and ventral hernia repair on 40 patients, respectively. There were no statistically significant differences in operation time and intraoperative bleeding between the groups. There were 11 postoperative complications (CD ≧ Ⅱ), four cases with BCs, and seven cases with non-BCs (four cases of pneumonia, two cases of seroma, and one case of arrhythmia). The incidence of postoperative complications (CD ≧ Ⅱ) and postoperative BC was not significantly different. There was no TC in the whole cohort. 

**Table 2:** Factors concerning operative procedures and postoperative morbidity BC: bleeding complication, cAPT: continued single aspirin therapy, Intra-RBC Transf: intraoperative red blood cell transfusion, TAPP: transabdominal preperitoneal approach, TC: thromboembolic complication, TEP: totally extraperitoneal approach ^※^Postoperative complication (Clavien-Dindo ≧ Ⅱ)

Factors	Total (n = 396)	cAPT (n = 118)	non-APT (n = 278)	p-value
Type of surgery: TAPP, n	311	89	222	0.3258
Type of surgery: TEP, n	45	15	30	0.5818
Type of surgery: Ventral hernia repair, n	40	14	26	0.4480
Operation time (minutes), median (range)	111 (89-149)	109 (89-145)	112 (90-151)	0.5282
Intraoperative bleeding (mL), median (range)	5 (4-10)	5 (4-5)	5 (4-10)	0.4161
Intra-RBC Transf, n (%)	0 (0.0)	0 (0.0)	0 (0.0)	-
Postoperative BC, n (%)^※^	4 (1.0)	1 (0.9)	3 (1.1)	0.8330
Postoperative TC, n (%)^※^	0 (0.0)	0 (0.0)	0 (0.0)	-
Postoperative complication^※^	11 (2.8)	2 (1.7)	9 (3.2)	0.3929
Postoperative hospital stays (day), median (range)	4.6 ± 2.5	4.0 (3-6)	4.0 (3-5)	0.3792

Postoperative bleeding complications

BC occurred in four patients overall. Table 3 summarizes these four postoperative BC cases. Three out of four BC patients were in the ATT group, and one was in the non-ATT group. All three BC cases in the ATT group received heparin bridging or DOAC replacement. All of the BC occurred after the ATT was reinstated. Only one BC case occurred in the cAPT group, which also involved heparin bridging. We observed minor bleeding in the intraperitoneal, subcutaneous, and rectus muscles, and we conservatively controlled all of these BC cases without surgical intervention.

**Table 3:** Postoperative bleeding complication cases in this study ACT: anticoagulation therapy, APT: antiplatelet therapy, ATT: antithrombotic therapy, cAPT: continued single aspirin therapy, CD: Clavien-Dindo, DAPT: dual antiplatelet therapy, DOAC: direct-acting oral anticoagulants, TAPP: transabdominal preperitoneal approach, TEP: totally extraperitoneal approach

Case no.	Type of surgery	Group	APT	DAPT	ACT	cAPT	Heparin bridging	Convert to DOAC	Site of bleeding	CD grade
1	TAPP	ATT	+	+	+	-	+	-	Intraperitoneal bleeding	Ⅱ
2	TEP	ATT	+	-	+	+	+	-	Subcutaneous hematoma	Ⅱ
3	TAPP	ATT	-	-	+	-	-	+	Rectus muscle hematoma	Ⅲ
4	Ventral hernia repair	non-ATT	-	-	-	-	-	-	Intraperitoneal bleeding	Ⅱ

Table 4 displays the results of univariate and multivariate analyses of postoperative BC in the cohort. Multiple ATT used, ACT used, and heparin or DOAC replacement were associated with postoperative BC in the univariable analyses. In the multivariable analysis, heparin or DOAC replacement (p < 0.0029, odds ratio (OR) = 32.6) was independently and significantly associated with postoperative BC. The continuation of preoperative aspirin was not associated with postoperative BC. 

**Table 4:** Univariate and multivariate analyses of postoperative bleeding complications in the whole cohort ACT: anticoagulation therapy, APT: antiplatelet therapy, ASA-PS: American Society of Anesthesiologists-Physical Status, ATT: antithrombotic therapy, CABG: coronary artery bypass graft, DAPT: dual antiplatelet therapy, DOAC: direct-acting oral anticoagulants, PCI: percutaneous coronary intervention, TIA: transient ischemic attack

Variables	No	Univariate		Multivariate analysis		
		Present (%)	p-value	Odds ratio	95% CI	p-value
Age > 75, n	186	3 (1.6)	0.2589	-	-	-
ASA-PS ≧ 3, n	94	1 (1.1)	0.9524	-	-	-
Concurrent diseases: Hypertension, n	265	3 (1.1)	0.7299	-	-	-
Concurrent diseases: Diabetes mellitus, n	56	1 (1.8)	0.5310	-	-	-
Hx of cerebral infarction/TIA, n	59	1 (1.7)	0.5685	-	-	-
Hx of PCI, n	92	1 (1.1)	0.9329	-	-	-
Hx of CABG, n	11	0 (0)	0.7340	-	-	-
Regular hemo/peritoneal dialysis	20	0 (0)	0.6429	-	-	-
CHA_2_DS_2_-VASc, score ≧2	322	4 (1.2)	0.3352	-	-	-
CHADS_2_ score≧2, n	221	4 (1.8)	0.0736	-	-	-
ATT used, n	187	3 (1.6)	0.2634	-	-	-
Multiple ATT, n	31	2 (6.5)	0.0016	1.8	0.16-41.8	0.6248
Continuation of APT, n	118	1 (0.9)	0.8330	-	-	-
ACT, n	80	3 (3.8)	0.0061	0.6	0.005-46.6	0.8373
DAPT, n	23	1 (4.4)	0.0991	-	-	-
Heparin or DOAC replacement, n	36	3 (8.3)	<0.0001	32.6	3.3-323	0.0029

## Discussion

In this study, we investigated bleeding and TCs associated with surgery for laparoscopic hernia surgery and the safety of continuing aspirin. Multivariate analysis showed that heparin or DOAC replacement was an independently and significantly risk factor for postoperative BC, and continuing preoperative aspirin therapy was not a risk factor for BC. No TC occurred in the whole cohort.

Fujikawa et al. [9] analyzed the outcomes of various types of laparoscopic digestive surgery in patients receiving ATT to prevent thromboembolism in the systematic review. In this systematic review, he described that patients with continued ATT or heparin bridging did not substantially increase the risk of hemorrhagic or TCs during or after these procedures as compared to those without ATT or with interrupted APT. Furthermore, we have published that patients who continue perioperative aspirin monotherapy for high-thromboembolic-risk patients (“Kokura Protocol”) can safely perform laparoscopic digestive surgery [7,8]. 

There is so far a paucity of data regarding the risk and safety associated with laparoscopic hernia surgery in patients who receive ATT. The optimal perioperative management of ATT in cases of laparoscopic hernia surgery remains debatable. In this study, we investigated the safety and feasibility of laparoscopic hernia surgery in patients taking ATT, especially those with a preoperative continuation of single antiplatelet therapy (cAPT). In terms of patient background characteristics, patients receiving antithrombotic drugs were treated for cardiovascular illness and cerebral infarction disease compared to non-ATT groups and had poorer ASA-PS than those who were not prescribed such medications. 

Hernia surgery is usually less invasive and generally carries a lower risk of bleeding. Postoperative bleeding occurred in 0.3-5% of hernia operations [10-13]. Even though up to 50% of patients undergoing laparoscopic hernia surgery regularly received ATT, postoperative BCs (CD ≧ Ⅱ) in this study were 1.0% in all cases and 1.6% limited to the ATT group. This is comparable to previous literature [10-13]. All BC cases in the ATT group were treated with heparin bridging or DOAC replacement. Only one case in the cAPT group developed BC, which was also a heparin bridging case. Taking ATT preoperatively, including preoperative continuation of aspirin monotherapy, did not increase postoperative BC (CD ≧ Ⅱ).

A recent study from the Herniamed Registry of 82,911 patients undergoing open or endoscopic (TAPP, TEP) inguinal hernia repair reported a significantly higher postoperative bleeding rate of 3.91% in patients receiving ATT or with coagulopathy, compared with 1.12% in controls (in the non-ATT group) with a total rate of 1.42% [12]. Another study revealed a hematoma rate of 1.4% [14]. On the other hand, a systematic review that examined the risk and safety of perioperative antiplatelet and ACT in patients undergoing elective inguinal hernia repair indicated that there is no need to stop APT for both open and laparoscopic inguinal hernia repair; instead, the continuation or cessation of anticoagulation with warfarin should be tailored on an individual basis due to the complexity of each patient’s condition and the available evidence [15]. 

We have managed high-thromboembolic-risk patients with continued perioperative aspirin monotherapy (“Kokura Protocol”) [7,8]. There are some reports that discontinuing APT increases the risk of major ischemic cardiovascular events by about threefold [16] and by 37% [17]. Studies on hernia repair have rarely reported postoperative cardiovascular events. Ong et al. [18] reported that 2.4% of patients undergoing elective inguinal hernia repair who discontinued aspirin preoperatively developed cardiovascular thrombotic events. Our investigation has not resulted in any TCs. Once TC occurs, it can be fatal or cause severe impairment with a poor prognosis [19]. Therefore, we think it is preferable not to discontinue APT prior to surgery.

This study has several limitations. First, this is a retrospective observational design from a single institution, so there may be potential biases such as surgeon experience, background, or patient selection bias. We conducted the multivariate analysis to more effectively manage confounding variables; however, it remains possible that unknown confounding factors exist and reduce the significance of the findings. Second, this study had a small sample size, and laparoscopic hernia surgery generally confirmed low event rates; these factors may restrict generalizability. Third, our study only focused on the safety of hernia surgery under laparoscopic. Therefore, we have not determined the safety and feasibility of other open techniques for hernia repair. Fourth, our findings might not apply to all institutions because our institution is a high-volume tertiary referral hospital for surgical patients receiving antithrombotic treatment. Prospective multi-institutional studies or follow-up investigations will minimize this limitation. The investigators will continue to analyze additional cases to determine the optimal management of perioperative ATT cases for laparoscopic hernia surgery in this high-risk patient population.

## Conclusions

We can safely and feasibly perform laparoscopic hernia surgery with continuation of perioperative aspirin monotherapy without increasing the incidence of bleeding events. However, patients who received preoperative heparin bridging or DOAC replacement may increase the incidence of postoperative BC (CD ≧ Ⅱ).
